# Surgical outcomes in adolescents and adults with anomalous aortic origin of a coronary artery

**DOI:** 10.3389/fcvm.2024.1489303

**Published:** 2024-11-22

**Authors:** Baotong Li, Li Ding, Hansong Sun

**Affiliations:** ^1^ State Key Laboratory of Cardiovascular Disease, Department of Adult Cardiac Surgery, Fuwai Hospital, National Center for Cardiovascular Disease, Chinese Academy of Medical Science, Peking Union Medical College, Beijing, China; ^2^ Department of Cardiovascular Surgery, Union Hospital, Tongji Medical College, Huazhong University of Science and Technology, Wuhan, China

**Keywords:** anomalous aortic origin of a coronary artery, coronary artery, surgical treatment, CABG, congenital cardiac anomaly

## Abstract

**Background:**

Anomalous aortic origin of a coronary artery (AAOCA) is associated with an increased risk of myocardial ischemia and sudden cardiac death. This study aims to evaluate the medium-term outcomes of surgical repair for AAOCA and to introduce a novel off-pump technique for anomalous coronary artery reimplantation.

**Methods:**

We retrospectively reviewed the medical records of 12 patients aged 12 years and older who underwent AAOCA surgery at Fuwai Hospital between 2009 and 2016.

**Results:**

The median age at surgery was 26 years (range, 13–57 years). Patients with an anomalous left coronary artery from the right sinus (ALCA-R) were significantly younger than those with an anomalous right coronary artery from the left sinus (ARCA-L) (*P* < 0.001). During a median follow-up of 13 years (range, 8–15years), 11 patients had widely patent repaired coronary arteries, with the exception of one patient (case 5) who experienced occlusion of the left internal mammary artery graft 1 year post-CABG. The incidence of postoperative cardiac-type symptoms (angina, syncope or dyspnea) was higher in ALCA-R patients compared to ARCA-L patients. Patch angioplasty using a pulmonary artery patch and RCA reimplantation without cardiopulmonary bypass yielded satisfactory medium-term outcomes.

**Conclusions:**

Compared with ARCA-L, the incidence of postoperative cardiac-type symptoms was higher in ALCA-R patients. Properly selected surgical procedures can lead to successful outcomes in patients with AAOCA. Patch angioplasty with a pulmonary artery patch and RCA reimplantation without cardiopulmonary bypass are viable and effective surgical options. CABG may not be the preferred surgical approach for AAOCA.

## 1 Introduction

Anomalous aortic origin of a coronary artery (AAOCA) is observed in approximately 0.14% of the population (1–4), with the most common variations being an anomalous right coronary artery originating from the left sinus (ARCA-L) and an anomalous left main coronary artery originating from the right sinus (ALCA-R), especially when an interarterial course (IAC) exists between the aorta and pulmonary artery. AAOCA is linked to a heightened risk of myocardial ischemia, infarction, and infrequent cause of sudden cardiac death (SCD). Surgical intervention is typically pursued to mitigate ischemic events and includes procedures such as coronary artery bypass grafting (CABG), patch enlargement of the proximal coronary artery, reimplantation into the correct sinus, and unroofing, particularly in cases involving an intramural coronary course (1).

Despite the known benefits and limitations of each surgical technique, there is ongoing debate regarding the optimal choice of procedure, which is highly individualized. This study seeks to review our institutional experience with AAOCA and evaluate the medium-term outcomes following surgical repair. We conducted a retrospective observational analysis of 12 AAOCA patients treated at our institution, including the first documented case of off-pump reimplantation for an ARCA-L.

## 2 Patients and methods

### 2.1 Patients and follow-up

Surgical records from Fuwai Hospital in Beijing, China, were collected and retrospectively reviewed. Candidates were patients aged 12 and older. From 2009–2016, 12 patients with AAOCA received surgical treatment as part of this study. The study was approved by the Ethics Committee of Fuwai Hospital (approval number: 2022–1874) and conducted following the Declaration of Helsinki and approved guidelines. The consent of patients has been obtained during follow-up. Follow-up was conducted through online or phone interviews and outpatient records. The median follow-up period was 13 years (range, 8–15years), with a 100% follow-up rate.

### 2.2 Surgical technique

All procedures were performed through a median sternotomy. With the exception of case 10, all surgeries utilized cardiopulmonary bypass, aortic cross-clamping, and intermittent antegrade cold-oxygenated blood cardioplegia. The surgical techniques included intramural coronary artery unroofing, coronary artery reimplantation, patch angioplasty, and CABG. Our choice of surgical technique was individualized, based on patients' anatomical features, age, and imaging findings. For patients with significant narrowing or acute-angle takeoff, we preferred unroofing or reimplantation. For patients with limited proximal coronary diameter, patch angioplasty provided effective enlargement. Considering patient age and expected life span, we favored CABG for older patients or those with multiple risk factors.

#### 2.2.1 Unroofing

The intramural coronary course was evaluated by carefully passing graduated coronary probes from the native coronary ostium through the intramural segment until the probe emerged within the coronary artery, surrounded by epiaortic fat. The aortic layer separating the intramural coronary lumen from the aortic lumen was completely excised. A neo-ostium was constructed using interrupted 7-0 polypropylene sutures to secure full-thickness bites of the coronary artery to all layers of the aortic wall. When the intramural course passed behind the aortic valve commissure, unroofing was performed on either side of the commissure, leaving it intact and reinforced with a pledgeted suture.

#### 2.2.2 Reimplantation

After the proximal coronary artery was carefully identified and mobilized, it was transected at its emergence from the aortic wall, and the proximal end was oversewn. A new proximal site was created within the appropriate sinus of Valsalva using an aortic punch, and an end-to-side anastomosis was performed using continuous 5-0 polypropylene sutures. Notably, in case 10, RCA reimplantation was successfully performed without cardiopulmonary bypass.

#### 2.2.3 Patch angioplasty

Three patients with ALCA-R underwent patch angioplasty using either an autologous pericardial patch (cases 4 and 6) or a pulmonary artery patch (case 7). The aorta was transected, and an incision was made into the ostium of the anomalous coronary artery, extending into the coronary artery itself. A triangular patch was sutured into the incision to enlarge the diameter of the proximal coronary artery trunk, and the aortic anastomosis was completed by incorporating the top edge of the triangular patch into the suture line.

#### 2.2.4 CABG

CABG was performed using either pedicled internal mammary artery grafts or saphenous vein aortocoronary bypass grafts, without proximal ligation of the native coronary artery. Intraoperative graft blood flow was measured using transit-time flow measurement. Case 5 underwent CABG with two grafts: left internal mammary artery to left anterior descending artery and saphenous vein to obtuse marginal artery. Case 8 received CABG with right internal mammary artery grafts to the right main coronary artery, and case 12 received saphenous vein aortocoronary bypass grafts to the posterior descending artery.

### 2.3 Medical therapy

All the patients received aspirin 100 mg per day for 1 year postoperatively.

### 2.4 Statistical analysis

Continuous variables are reported as mean ± SD or median (interquartile range), while categorical variables are expressed as percentages. Preoperative characteristics between groups were compared using chi-square tests and *t*-tests. *P*-values < 0.05 were considered statistically significant. Statistical analyses were conducted using SPSS 20.0 (SPSS, Inc., Chicago, IL) and GraphPad Prism 5 (GraphPad Software Inc, La Jolla, Calif). Cumulative event rates were calculated using the Kaplan-Meier method, and outcome curves were compared using the Log-Rank test.

## 3 Results

**Figure 1:**
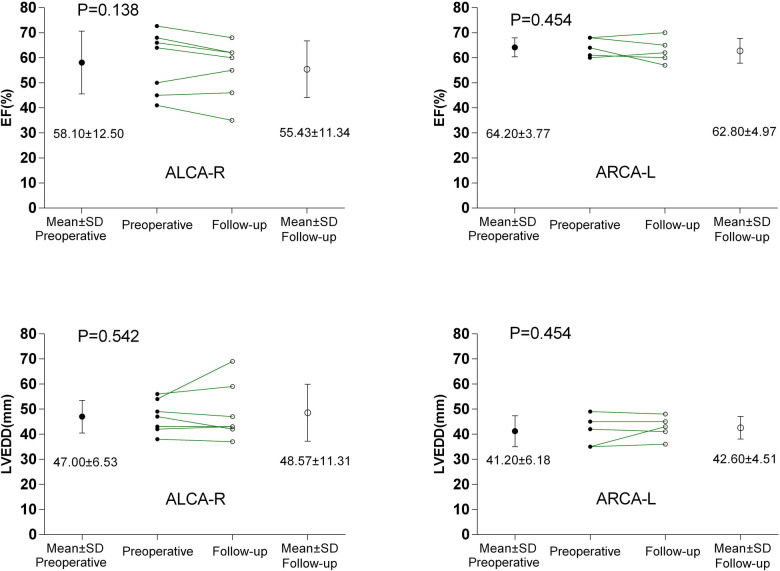
Comparisons between preoperative and postoperative EF and LVEDD. ALCA-R, anomalous left coronary artery arising from the right sinus; ARCA-L, anomalous right coronary artery arising from the left sinus; EF, left ventricular ejection fraction; LVEDD, left ventricular end-diastolic dimension.

The preoperative characteristics of the patients and the surgical procedures performed are summarized in Table 1. The median age at the time of surgery was 26 years (range, 13–57 years). Five patients (41.7%) with ARCA-L were older than 32 years, while seven patients (58.3%) with ALCA-R were younger than 32 years. All 12 patients presented with symptoms of angina, dyspnea, or syncope prior to surgery, and three patients with ALCA-R had experienced preoperative myocardial infarctions. All patients had high-risk anatomical features associated with AAOCA, including a slit-like ostium, acute angle of takeoff, and interarterial course. A comparison of the clinical characteristics between ALCA-R and ARCA-L patients is shown in Table 2. There were no significant differences between ALCA-R and ARCA-L in terms of sex or preoperative left ventricular ejection fraction (EF). However, patients with ALCA-R were significantly younger (*P* < 0.001) and appeared to have a longer duration of symptoms and larger preoperative left ventricular end-diastolic dimension (LVEDD), although these differences were not statistically significant. No significant differences were observed between preoperative and postoperative EF or LVEDD in either group (Figure 1).

**Table 1:** Patient characteristics and follow-up.

ID.	Coronary anatomy	Age at surgery (years) and sex	Presenting symptoms (duration before surgery, months)	Procedure	Concomitant procedures	Follow-up time, years	Presenting symptoms or activity limitation at follow-up
1	ALCA-R	13, male	Angina, STEMI (36)	Reimplantation	No	9	Yes
2	ALCA-R	14, female	Angina, syncope, STEMI (12)	Unroofing	No	14	Yes
3	ALCA-R	15, male	Syncope (6)	Unroofing	No	10	No
4	ALCA-R	17, male	Angina, syncope, STEMI (108)	Patch ostioplasty	No	15	Yes
5	ALCA-R	17, female	Angina, syncope (48)	CABG	No	12	Yes
6	ALCA-R	20, male	Angina, syncope (24)	Patch ostioplasty	No	12	No
7	ALCA-R	31, female	Angina (1)	Patch ostioplasty	No	8	No
8	ARCA-L	33, male	Angina (1)	CABG	No	14	No
9	ARCA-L	37, male	Angina (10)	Reimplantation	No	13	No
10	ARCA-L	47, female	Dyspnea (2)	Reimplantation	No	12	No
11	ARCA-L	55, female	Angina (12)	Unroofing	Repair of ASD	13	No
12	ARCA-L	57, female	Angina (12)	CABG	No	15	No

ALCA-R, anomalous left coronary artery arising from the right sinus; ARCA-L, anomalous right coronary artery arising from the left sinus; STEMI, ST segment elevation myocardial infarction; CABG, coronary artery bypass graft; ASD, atrial septal defect.

**Table 2:** Comparison of clinical characteristics between ALCA-R and ARCA-L.

	ALCA-R (*n* = 7)	ARCA-L (*n* = 5)	*P*
Age at surgery, years	18.14 ± 6.12	45.80 ± 10.64	<0.001
Sex (male), *n* (%)	4 (57.1)	2 (40.0)	>0.999
Average duration of symptoms, months; Median (Interquartile range)	24 (12–42)	10 (2–12)	0.073
Preoperative EF	58.10 ± 12.50	64.20 ± 3.77	0.261
Preoperative LVEDD	47.00 ± 6.53	41.20 ± 6.18	0.152
Follow-up EF	55.43 ± 11.34	62.80 ± 4.97	0.207
Follow-up LVEDD	48.57 ± 11.31	42.60 ± 4.51	0.294

ALCA-R, anomalous left coronary artery arising from the right sinus; ARCA-L, anomalous right coronary artery arising from the left sinus; EF, left ventricular ejection fraction; LVEDD, left ventricular end-diastolic dimension.

Of the 12 patients, three underwent unroofing, three underwent CABG, three underwent patch angioplasty of the proximal coronary artery, and three underwent reimplantation. There were no operative mortalities or early morbidities (death in 30 days after surgery). The median hospital stay was 9 days (range, 7–12 days).

**Figure 2:**
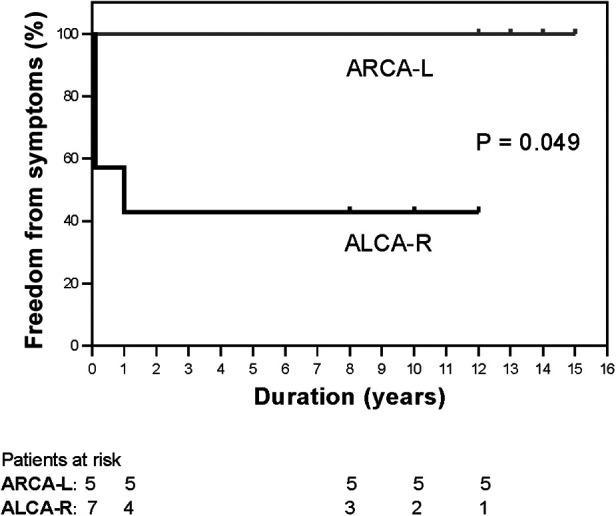
Kaplan-Meier curves for freedom from cardiac-type symptoms between ALCA-R (black lines) and ARCA-L (gray lines). ALCA-R, anomalous left coronary artery arising from the right sinus; ARCA-L, anomalous right coronary artery arising from the left sinus.

**Figure 3:**
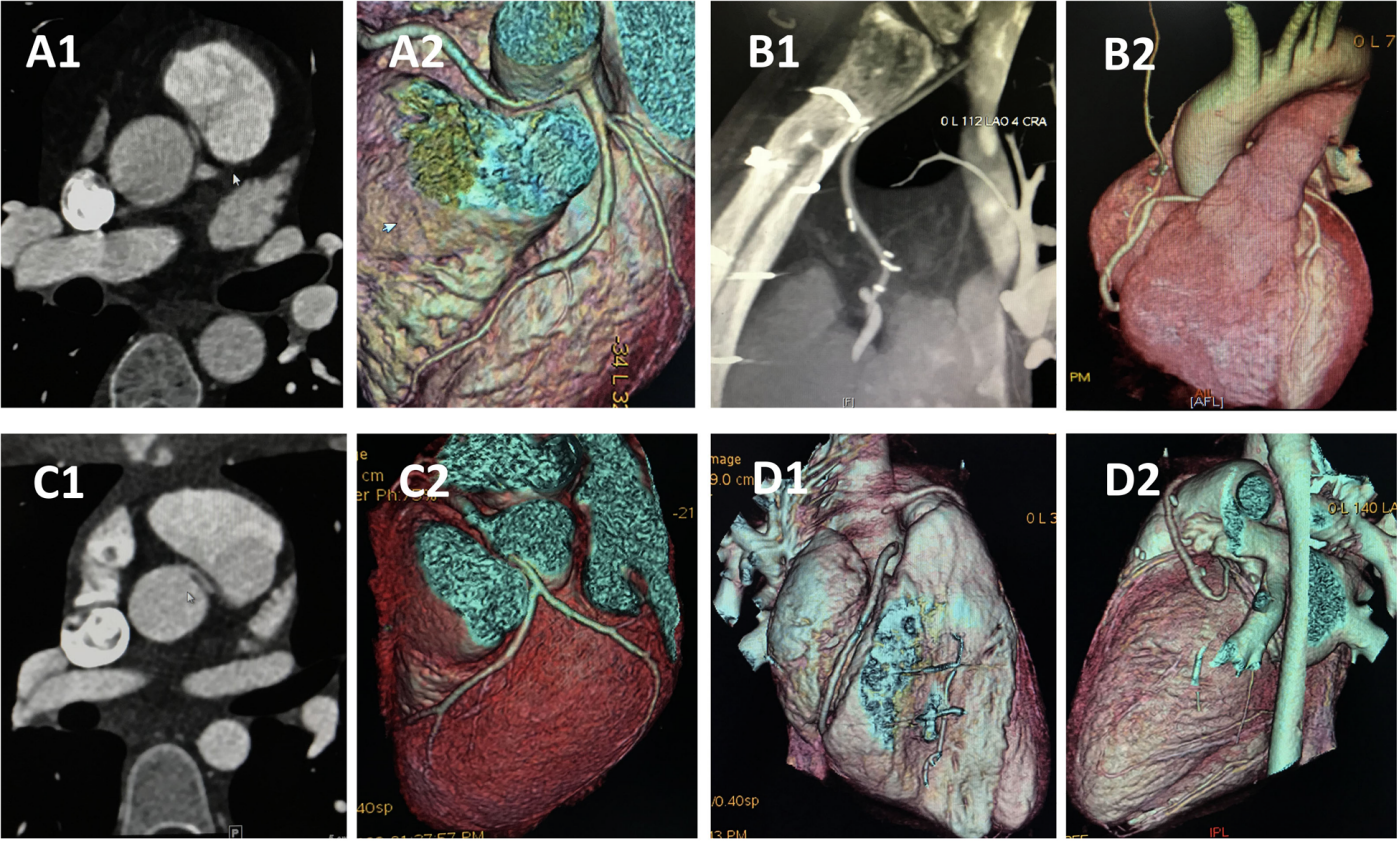
CCTA of case 5 and case 8 underwent CABG. Case 8: **(A)** Preoperative images showing anomalous right coronary artery arising from the left sinus. **(B)** Follow-up images showing right internal mammary artery grafts to right main coronary artery without visible stenosis. Case 5: **(C)** Preoperative images showing anomalous left coronary artery arising from the right sinus. **(D)** Follow-up images demonstrated that the left internal mammary artery was obstructed 14 monthes after operation. CCTA, coronary computed tomography angiography; CABG, coronary artery bypass graft.

**Figure 4:**
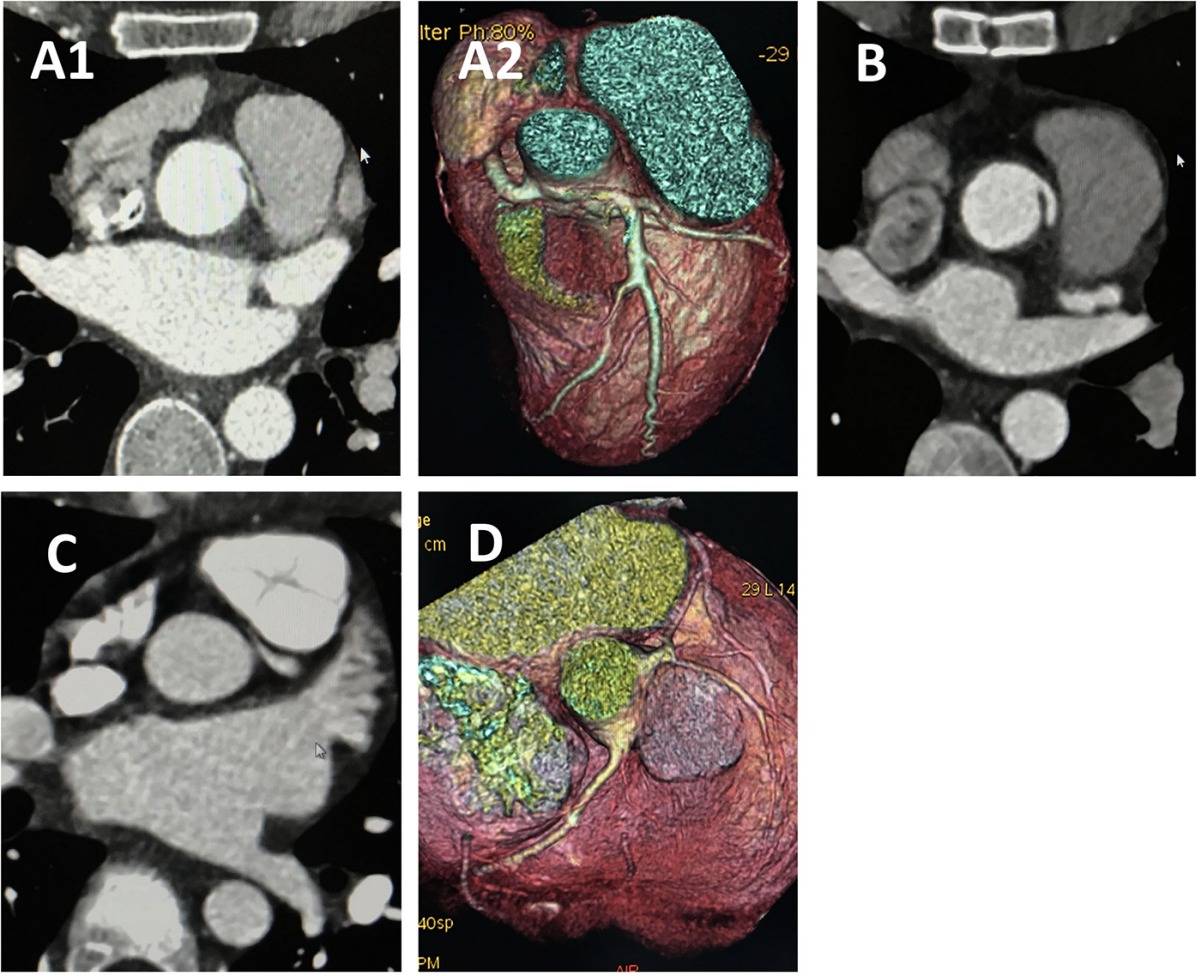
CCTA of case 7 and case 4 underwent angioplasty. Case 7 underwent angioplasty with pulmonary artery patch: **(A)** Preoperative images showing anomalous left coronary artery arising from the right sinus. **(B)** Follow-up images showing proximal left main coronary artery without visible stenosis. Case 4 underwent angioplasty with pericardial patch: **(C)** Preoperative images showing anomalous left coronary artery arising from the right sinus. **(D)** Follow-up images showing proximal left main coronary artery without visible stenosis. CCTA, coronary computed tomography angiography.

**Figure 5:**
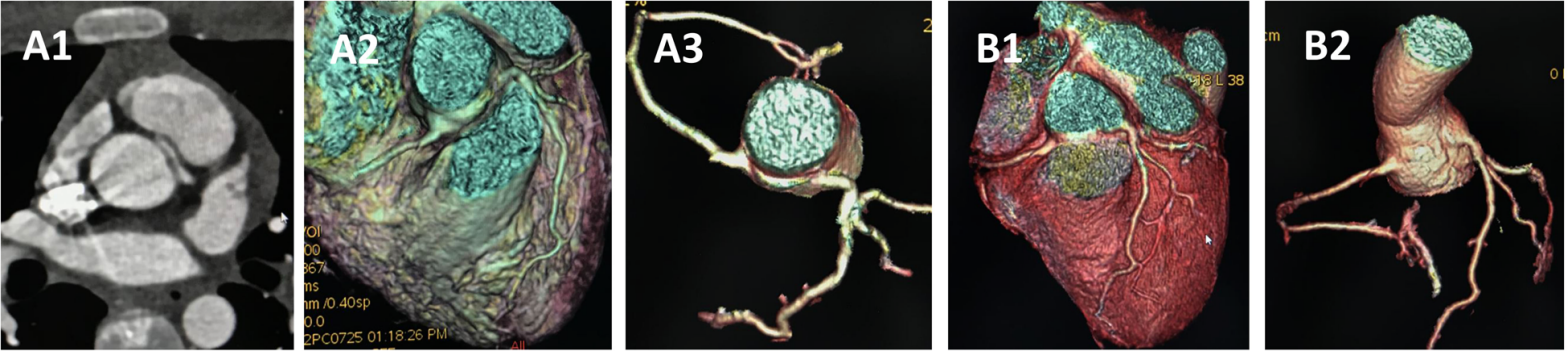
CCTA of case 2 underwent unroofing. **(A)** Preoperative images showing anomalous left coronary artery arising from the right sinus. **(B)** Follow-up images showing proximal left main coronary artery without visible stenosis. CCTA, coronary computed tomography angiography.

**Figure 6:**
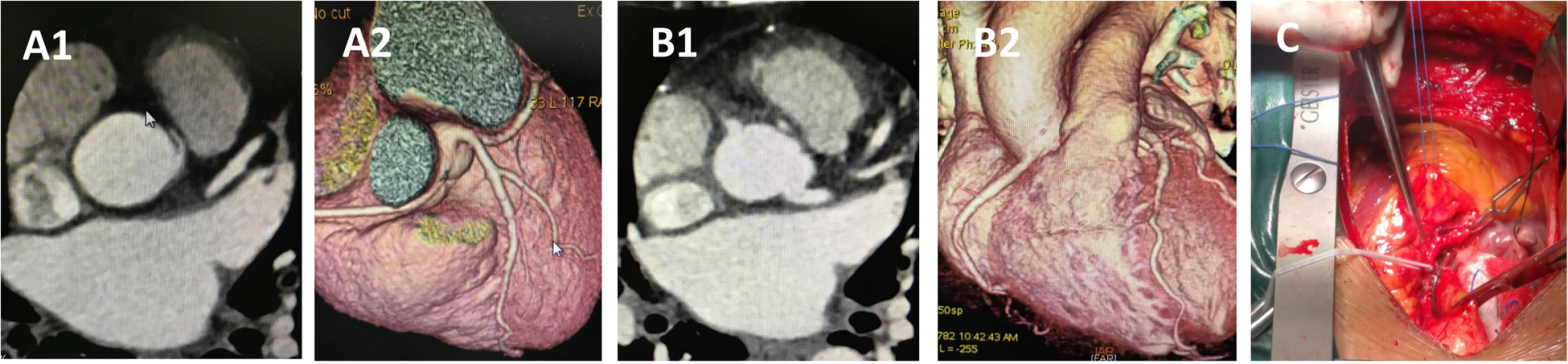
CCTA of case 10 underwent reimplantation. **(A)** Preoperative images showing anomalous right coronary artery arising from the left sinus. **(B)** Follow-up images showing proximal right main coronary artery without visible stenosis. **(C)** Intraoperative picture showing the reimplantation procedure without cardiopulmonary bypass. CCTA, coronary computed tomography angiography.

The median follow-up period was 13 years (range, 8–15years), with a 100% follow-up rate. All patients were alive at the most recent evaluation; however, four patients (33.3%) with ALCA-R reported experiencing cardiac-type symptoms (angina, syncope,dyspnea) postoperatively, most commonly angina and dyspnea. For this study, cardiac-type symptoms were defined as those occurring 3 months or longer after surgical repair. Of these four patients, two were restricted from physical activity. Compared with ARCA-L, the incidence of postoperative cardiac-type symptoms was higher in ALCA-R patients (*P* = 0.049) (Figure 2). All patients underwent follow-up coronary angiography or computed tomography angiography (CCTA) (Figures 3–6). All repaired coronary arteries were widely patent, except for case 5, in which the left internal mammary artery graft was found to be occluded 14 months postoperatively.

Case 10, a 47-year-old woman, presented with dyspnea for 2 months before surgery. Coronary angiography and CCTA revealed that the RCA originated from the left coronary sinus with an IAC between the aorta and pulmonary artery. The procedure was performed through a median sternotomy without cardiopulmonary bypass (Supplementary Video S1). After determining that the origin segment of the RCA was of sufficient length, and following adequate mobilization of the RCA and 1 min of ischemic preconditioning, the aorta was partially cross-clamped, and the RCA was clamped with vascular clips at both the emergence and a distal point. The RCA was then transected at a normal point proximal to the stenosis. A 5 mm hole was punched into the right coronary sinus, and the RCA was reimplanted in an end-to-side fashion using continuous 5-0 polypropylene sutures. The stub of the RCA was closed by oversewing it to the aortic wall using 7-0 polypropylene sutures. The total cross-clamping time for the RCA was 5 min. Hemodynamics were stable throughout the procedure, and the patient reported no postoperative complaints. Follow-up CT at 6 months confirmed that the repaired coronary artery remained patent. To the best of our knowledge, this is the first reported case of RCA reimplantation without cardiopulmonary bypass.

## 4 Discussion

Studies of young athletes have identified anomalous coronary arteries (ACAs) as the second leading cause of sudden cardiac death, with anomalous left coronary artery from the right sinus (ALCA-R) and associated interarterial course (IAC) being the most common contributors (5, 6). Diagnosing these anomalies poses a significant challenge, as survivors of aborted cardiac death are frequently misdiagnosed with primary ventricular arrhythmia instead of an anomalous coronary origin (7). Although echocardiography can reveal an anomalous origin, coronary anatomy is rarely the primary focus in examinations of young individuals (8). In our study, patients with ALCA-R were notably younger and experienced a longer duration of symptoms before seeking medical care, despite severe cardiac symptoms. Reports also suggest that patients with anomalous right coronary artery from the left sinus (ARCA-L) are more likely to survive undiagnosed into adulthood compared to those with ALCA-R (9), aligning with our findings. Additionally, postoperative cardiac symptoms were more prevalent in ALCA-R patients, underscoring the need for heightened vigilance when assessing young patients with ischemic symptoms. The higher incidence of postoperative cardiac-type symptoms in ALCA-R patients may relate to incomplete revascularization, patient anatomy, and specific surgical challenges. While most postoperative symptoms were manageable with medication, some patients reported limited physical activity.

The 2018 American College of Cardiology (ACC) and American Heart Association (AHA) guidelines recommend revascularization for anomalous aortic origin of a coronary artery (AAOCA) patients with documented ischemia as a Class I indication (10). Various surgical approaches have been documented, with the unroofing technique being the most frequently performed.

### 4.1 Unroofing

First described by Mustafa and colleagues (11), unroofing of the intramural coronary artery segment has become the standard intervention for AAOCA. To minimize residual stenosis risk, the unroofing is extended until epiaortic fat is exposed. The neo-ostium is reconstructed with full-thickness sutures between the coronary artery and the aortic wall, with patency confirmed via direct inspection and insertion of a large coronary probe (12). Mainwaring et al. reported a substantial series of unroofing procedures without early or midterm complications, consistent with our findings (13). However, other studies have indicated early complications, such as the need for coronary artery bypass grafting (CABG) when unroofing fails to address an interarterial right coronary artery (RCA) (14).

### 4.2 Reimplantation

This approach excludes the intramural component and achieves anatomical correction, avoiding long-term complications related to coronary artery bypass grafts (15). Reimplantation is particularly recommended when coronary ostia are located near the aortic valve commissures (16, 17). Timothy's work demonstrates that reimplantation results in a stenosis-free origin positioned normally (18). In our study, reimplanted coronary arteries showed no acute angulation or compression by the pulmonary artery. Notably, we report the first case of RCA reimplantation without cardiopulmonary bypass, showing that with skilled surgical techniques, this method is a viable and effective alternative. The main challenge in reimplantation is ensuring the coronary artery length is optimal to avoid kinking. Precise mobilization of the distal segment and accurate length estimation are essential for success.

### 4.3 Patch angioplasty

Proximal patch angioplasty for the anomalous coronary artery is versatile, applicable across various coronary ostial configurations and narrowing lengths. This technique enlarges the ostium, increases the proximal coronary artery diameter, and improves the acute takeoff angle. Midterm patency has been satisfactory with autologous patch material in both children and adults (8, 19). Combined with pulmonary artery translocation, patch angioplasty provides a physiological repair strategy addressing multiple ischemia mechanisms (20). However, this method may not be feasible when the anomalous coronary shares a common ostium with another coronary artery or if repair requires aortic valve commissure detachment (7, 21). Gaudin et al. reported pericardial patch repair in five AAOCA patients, with one requiring reoperation due to a patch aneurysm six months postoperatively, suggesting caution in using this approach (22). To our knowledge, we present the first case of ALCA-R treated via pulmonary artery patch angioplasty. Without proximal pulmonary artery translocation, this technique yielded satisfactory outcomes.

### 4.4 CABG

Although CABG is preferred by some (23), early graft failure often occurs in AAOCA patients due to competitive flow causing graft occlusion or atresia (14). Consistent with the literature, one of the three patients in our study who underwent CABG had an occluded graft on follow-up CT. For younger patients, internal mammary artery grafts are recommended for better long-term patency (24). However, without proximal ligation, arterial grafts commonly fail due to competitive flow. Sabik et al. reported that CABG with a right internal mammary artery graft was prone to failure without proximal ligation (25). While proximal coronary artery ligation can prevent competitive flow, it is not always feasible due to risks like hypoperfusion syndrome, ischemia, or even mortality (26, 27). Given our limited sample size, evaluating the overall efficacy of CABG in AAOCA remains challenging. Although we observed graft occlusion in some CABG cases, the additional step of autologous conduit harvesting may increase surgical trauma and potential complications. Therefore, a less invasive alternative could be advantageous for certain patients, but larger studies are needed to validate this hypothesis.

## 5 Limitations of the study

This study presents retrospective data from a single center, subject to all inherent limitations of this design. The small sample size may have introduced type II statistical errors. A well-powered trial to evaluate optimal AAOCA management would be valuable in confirming these findings. The retrospective nature of the study and the absence of a control group (e.g., patients managed conservatively or non-surgically) make it difficult to assess whether surgical intervention, particularly the novel off-pump technique, offers superior outcomes compared to non-surgical approaches or traditional surgery. Due to the shorter follow-up duration, this study has limitations in assessing the long-term durability of AAOCA repairs. We plan to continue following our patient cohort to gain additional insight into long-term patency and survival, especially for younger patients. Future studies should consider extended follow-up to better evaluate surgical durability and outcomes. Given the low incidence of AAOCA, single-center data limit generalizability. We propose future studies should aim to collaborate across multiple centers to increase sample size and enhance statistical power, thus providing more widely applicable insights.

## 6 Conclusion

Patients with ALCA-R were significantly younger and had a longer duration of symptoms before seeking medical attention, despite experiencing severe cardiac symptoms. Additionally, the incidence of postoperative cardiac-type symptoms was higher in ALCA-R patients. Given the association of AAOCA with an increased risk of myocardial ischemia and infarction, as well as the high incidence of postoperative cardiac-type symptoms, greater attention should be paid to young patients presenting with ischemic symptoms. The selection of the surgical procedure should be based on specific anatomical details and the surgeon's experience, as this can lead to successful outcomes in AAOCA repair. In our opinion, CABG may not be the optimal choice for AAOCA, but more evidence is needed. When anatomically feasible, unroofing, reimplantation, and patch angioplasty are viable alternatives for this congenital anomaly.

To the best of our knowledge, this study reports the first case of ALCA-R treated with pulmonary artery patch angioplasty and the first case of RCA reimplantation without cardiopulmonary bypass. Both techniques present promising alternative surgical options with satisfactory outcomes.

## Data Availability

The original contributions presented in the study are included in the article/Supplementary Material, further inquiries can be directed to the corresponding author.

## Supplementary material

Supplementary Video S1 RCA reimplantation without cardiopulmonary bypass (case 10). RCA, right coronary artery.
